# Using genetics to investigate the association between lanosterol and cataract

**DOI:** 10.3389/fgene.2024.1231521

**Published:** 2024-02-19

**Authors:** Munisa Hashimi, Hasnat A. Amin, Loukas Zagkos, Alexander C. Day, Fotios Drenos

**Affiliations:** ^1^ Department of Life Sciences, College of Health, Medicine and Life Sciences, Brunel University London, Uxbridge, United Kingdom; ^2^ Department of Epidemiology and Biostatistics, Imperial College London, London, United Kingdom; ^3^ Moorfields Eye Hospital, London, United Kingdom; ^4^ UCL Institute of Ophthalmology, London, United Kingdom

**Keywords:** cataract, lens transparency, anti-cataract drug, lanosterol, oxysterol

## Abstract

**Background:** Cataract is one of the most prevalent causes of blindness worldwide. Whilst surgery is the primary treatment for cataracts, it is not always an available option, particularly in developing countries. Non-surgical methods of treatment would increase treatment availability for more patients. Several studies have investigated how topical application of oxysterols, such as lanosterol, may break down aggregated proteins and restore lens transparency. However, the results are conflicting and inconclusive.

**Aim:** In this study, we focus on combining genetic evidence for associations between lanosterol related genetic variation and cataract to explore whether lanosterol is a potentially suitable drug treatment option.

**Method:** Using data from 45,449 available cataract cases from the UK Biobank, with participant ages ranging from 40–69, we conducted a genetic association study (GWAS) to assess the risk of cataract. Cataract cases were defined using diagnostic and operation codes. We focused on genetic variants in the lanosterol synthase gene region. We also compared our results with previously published genetic associations of phytosterol-to-lanosterol ratios. Finally, we performed a genetic risk score analysis to test the association between lanosterol within the cholesterol synthesis pathway and the risk of cataract.

**Results:** No statistically significant single nucleotide polymorphisms (SNPs) associations with cataract were observed in the gene region of lanosterol synthase at a multiple testing adjusted significance threshold of *p* < 0.05/13. The comparison between cataract risk and genetic association of 8 phytosterol-to-lanosterol GWAS results also showed no evidence to support lanosterol’s protective properties for cataract risk. No statistically significant association was found between the lanosterol within the cholesterol synthesis pathway genetic risk score and cataract outcomes (OR = 1.002 *p* = 0.568).

**Conclusion:** There was no evidence observed for genetic associations between lanosterol and cataract risk. Our results do not support lanosterol’s potential role in treating cataracts. Further research may be needed to address the effect of lanosterol on specific cataract subtypes.

## Introduction

Cataract is the opacification of the crystalline lens, causing a loss in clarity of vision. In the development of cataracts, protein build-up occurs when crystallin proteins are damaged, leading them to misfold and aggregate into insoluble clumps. With age, fibre cells lose their protective functions; therefore, mature fibre cells are unable to remove damaged proteins from the lens. Over time proteins become increasingly damaged, causing clouding in the lens and cataract formation (Makley et al., 2015).

Cataract is one of the leading causes of blindness globally (Flaxman et al., 2017). In 2015 it was estimated that 1 in 3 cases of blindness were due to cataract (Khairallah et al., 2015). In 2020, it was estimated that 78.8 million individuals worldwide had moderate or severe vision impairment due to cataract, with its prevalence increasing due to a growing and ageing population (Pesudovs et al., 2021). Currently, the only treatment is cataract surgery, whereby the cataract is removed and replaced with an artificial intraocular lens. This is typically a safe and successful operation (Davis, 2016); however, complications still occur and can be serious (Chan et al., 2010; Naeem et al., 2012). Complications in cataract-related surgeries include intraoperative complications such as posterior capsule rupture and postoperative complications such as cystoid macular edema, endophthalmitis and retinal detachment (Hong et al., 2015; Garg et al., 2017; Liu et al., 2017; Kim et al., 2019). The success rate of cataract surgery in developing countries is significantly lower compared to developed countries, with studies highlighting the need for improved quality and quantity of surgeries in regulated and safer settings (Bourne et al., 2003; Bourne et al., 2007).

Whilst there are no non-surgical treatments for cataracts, non-invasive options would significantly lessen the burden on public services where, globally, more than 26 million cataract surgeries are performed annually, with the volume of procedures growing at an annual rate of 3.1% (Chen et al., 2021). The prevalence of cataract surgery is increasing due to an ageing population and other environmental factors (Purola et al., 2022). Among all eye diseases, the blindness rate caused by cataracts in poor and remote regions is estimated to be greater than 50%, compared to 5% in developed countries. Across some African regions, access to cataract services is estimated to be a 10th of what is available for high-income countries (Chen et al., 2021).

Medication to treat cataract would lessen the need for surgery and avoid its associated complications, thus providing a safer alternative treatment that individuals can easily access in poor and remote regions.

Recent studies have identified oxysterols, such as lanosterol, as being able to restore the transparency of lenses affected by cataracts (Makley et al., 2015; Zhao et al., 2015; Molnar et al., 2019; Wang et al., 2022). In addition, studies have discovered that lanosterol was effective in redissolving aggregates of bound proteins and restoring lens clarity in human lenses (Qi et al., 2016; Chen et al., 2018).

Zhao et al. found that lanosterol reduced cataract severity and increased lens clarity in animals, specifically *in vitro* in rabbit cataractous lenses and *in vivo* in dogs (Zhao et al., 2015). Defects in lanosterol synthase (LSS), which synthesises lanosterol, have also been found to be associated with congenital cataracts in humans (Zhao et al., 2021). Following the Zhao et al. publication, lanosterol eyedrops have been marketed with claims to reverse the effects of cataracts, more commonly for the use in animals (Balashova et al., 2018). However, further research on lanosterols has questioned their effectiveness as a cataract treatment. Whilst Balashova et al. found evidence for stabilising rapidly progressive cataracts in a human patient using lanosterol eyedrops, Shanmugam et al. and Daszynski et al. found no evidence that lanosterol reverses lens opacification or affects lens protein solubilisation in cataractous human and animal lenses (Shanmugam et al., 2015; Balashova et al., 2018; Daszynski et al., 2019).

The production of drug-based treatments has historically been limited by the weak predictive efficacy found in preclinical experiments using cell, tissue, and animal models. Genomic data used for analysis is becoming an increasingly important part of drug development and benefit the process by facilitating target validation and being increasingly relevant to human biology rather than studying animal models of diseases (Spreafico et al., 2020). Single nucleotide polymorphisms (SNPs) associated with a gene affecting a protein of interest can be used as proxies to investigate a drug’s potential impact on the respective protein (Finan et al., 2017). The use of genetic evidence in selecting and assessing the efficacy of drug targets can significantly increase the likelihood of a drug reaching phase III of trials and entering the market (Nelson et al., 2015). Due to the recent success of genetic evidence, the use of genetic association results in drug development has become increasingly popular (Liou, 2014). However, genetic evidence on the effect of lanosterol on cataract has not yet been established.

In this study, using data from the UK Biobank we apply different genetic analysis approaches to investigate the relationship between lanosterol and cataracts. In brief, we tested if genetic variants in the lanosterol synthase gene region have a statistically significant association with cataract risk. We then extended our search to include genomic regions previously associated with lanosterol production and tested their association with cataract risk. Finally, we generated a genetic risk score using independent genetic variants previously associated with lanosterol, to test if their combined effect can provide evidence for lowering the risk of cataracts.

## Methods

### The UK Biobank

The UK Biobank (UKBB) is a large-scale population-based prospective study including over 500,000 participants within the United Kingdom. Participants were recruited between 2006 and 2010. The study aims to investigate risk factors, such as genetic predispositions and environmental exposures, for various diseases to find new ways to prevent and treat different conditions (UK Biobank, 2007; Allen et al., 2012). The age of participants at recruitment ranged from 40–69, with a mean of 56.5 years of age. Males represented 45.8% of the sample. Participants provided detailed environmental, lifestyle, and genetic data, including blood, urine and saliva samples, physical and functional measures, and access to their previous health records (Allen et al., 2012). Data was collected in 22 locations across England, Scotland, and Wales to prevent potential geographic bias and was anonymised (UK Biobank, 2007). To add to the phenotypic information available, comprehensive longitudinal follow-up examinations were undertaken (Sudlow et al., 2015). Across all cataract-related data fields in the UKBB, including self-reported data fields, 67,731 individuals are recorded to have been affected by cataract.

The UKBB approved the data used for this project under application 72850.

### Genotyping and imputation of the UKBB

DNA extracted from the blood samples was used for genotyping. A total of 488,377 participants were genotyped using two similar genotyping arrays. First, 49,950 participants from the UK Biobank Lung Exome Variant Evaluation (UK BiLEVE) study were genotyped using the Applied Biosystems UK BiLEVE Axiom Array with 807,411 markers. The remaining 438,427 participants were genotyped using the Applied Biosystems UK Biobank Axiom Array with 825,927 markers (Bycroft et al., 2018).

The samples were quality controlled (QC) during the DNA extraction and genotype calling stages, and relevant samples were removed. Further QC steps outlined by Bycroft et al. were applied to the data before performing the UKBB analyses; this included mismatched sex from baseline characteristics, which had self-reported sex (UKBB Data-Field 31) differing from genetic sex (UKBB Data-Field 22001). Further samples were removed from the analysis for the presence of sex chromosome aneuploidy (UKBB Data-Field 22019), poor quality genotypes identified via outliers in heterozygosity and missing rates (UKBB Data-Field 22027) (Bycroft et al., 2018). Participants excluded from the kinship inference process or with 10 or more third-degree relatives were also removed (UKBB Data-Field 22021). Individuals who requested data withdrawal from UKBB (as of April 2023) were removed from analyses.

To avoid population stratification that may produce false positive associations, samples were filtered to include only European individuals. Individuals’ ethnicity was established through self-reported data that they identified as “White British” and had appropriate genetic ancestry to match (UKBB Data-Field 22006).

The genotyped data was imputed using the Haplotype Reference Consortium, UK10K haplotype panel and 1,000 Genome Project (Bycroft et al., 2018). Inputted variants with an INFO score <0.8 or an EAF (effect allele frequency) < 0.01 were removed throughout the UKBB analysis. The imputation process has been described in further detail by Bycroft et al. (2018).

### Cataract definition

Individual level data was used to define cataract cases in the UKBB by identifying participants’ hospital inpatient records relating to diagnostic codes (UKBB Data-Field 41270—ICD-10: H25 Senile cataract, H26 Other cataract, H28 Cataract and other disorders of lens in diseases classified elsewhere and Q12.0 Congenital cataract; and UKBB Data-Field 41271—ICD-9: 7433 Congenital cataract and lens anomalies) and operation codes (UKBB Data-Field 41272—OPCS4: C75.1 Insertion of prosthetic replacement for lens NEC and C71.2 Phacoemulsification of lens). Any individuals with self-reported cataracts (UKBB Data-Field 6148) or self-reported cataract surgery (UKBB Data-Field 5324 and 20004), but no supporting diagnostic or operation codes, were removed to reduce the risk of misclassification in the cases and controls.

### Data for phytosterol traits

Genetic association estimates for the production of lanosterol from other phytosterols were obtained from Scholz et al. In this study, a genome-wide meta-analysis of the metabolism of phytosterols was performed using 9,758 individuals from six studies: KORA, LIFE-Adult, LIFE-Heart, LURIC and Sorbs. The conversion of phytosterols, which included brassicasterol, campesterol, sitosterol and stigmasterol, to lanosterol was described using the ratio of both total and free concentrations of each phytosterol-to-lanosterol ratio in reaction equilibria. In total, 8 phytosterol-to-lanosterol ratio traits were included (Scholz et al., 2022).

### Data for replication

Two cohorts were used to replicate any identified statistically significant associations:

1. GWAS summary statistics from FinnGen (R9) for cataract senile (59,522 cases and 312,864 controls) and cataract other (17,699 cases and 312,864 controls) (Kurki et al., 2023).
2. Multi-ethnic meta-analysed cataract GWAS summary statistics from Choquet et al. using UK Biobank and Genetic Epidemiology Research in Adult Health and Aging (GERA) cohorts. This included 585,243 individuals (67,844 cases and 517,399 controls) (Choquet et al., 2021).

### Statistical analysis

Analysis was performed using the statistical software R v4.0.5 (R Core Team, 2021), unless otherwise stated. PLINK v2.0 (https://www.cog-genomics.org/plink/2.0/) was used to identify independent SNPs and generate the genetic risk scores across the 8 phytosterol-to-lanosterol ratio traits.

### Genome-wide association study

Genetic associations for cataract development were obtained through a GWAS using the previously described cataract phenotype. The cataract GWAS was conducted using REGENIE v3.2.8. REGENIE is a C++ programme used to conduct whole genome regression modelling of large genome wide association studies, using a mixed-model-based approach (Mbatchou et al., 2021). Prior to performing a GWAS using REGENIE, additional QC steps were taken for the genotype input file using PLINK v2.0. QC removed SNPs with minor allele frequency (MAF) < 0.01, minor allele count (MAC) < 100, genotype missingness >0.1, Hardy-Weinberg equilibrium *p*-value >10^–15^, and samples with >0.1 missingness.

REGENIE is run across two steps:

1. A whole genome regression model is fitted using a subset of the available QCed SNPs that captures a portion of phenotypic variance and produces a set of genomic predictions.
2. Imputed SNPs are then tested for an association with the genomic predictions of Step 1, using a Firth logistic regression model.

Further information regarding the REGNIE QC and steps can be found at https://rgcgithub.github.io/regenie.

Following QC, 45,449 cases were identified, alongside 353,371 controls. A description of cases and controls can be found in Table 1. The covariates included were sex, age, agexsex, age squared and the first 10 principal components of the genetic data.

**TABLE 1:** UK Biobank case-control baseline characteristics by cataract status. If applicable, standard deviations are presented in round brackets.

	Females			Males		
	All	Cases	Controls	All	Cases	Controls
N	215,429	26,301	189,128	183,591	19,148	164,443
Mean Age (years)	56.61 (7.93)	62.62 (5.49)	55.78 (7.85)	57.06 (8.10)	62.63 (5.69)	56.41 (8.09)
Mean BMI (kg/m^2^)	27.05 (5.14)	27.68 (5.21)	26.96 (5.12)	27.85 (4.23)	28.23 (4.36)	27.81 (4.21)
Ever Smoked (%)	40.61	44.78	40.03	51.02	60.08	49.97
Have Diabetes (%)	3.38	7.19	2.85	6.45	13.19	5.67
Employed/Self-Employed (%)	54.28	29.13	57.78	59.98	35.96	62.77

### Approach 1: identifying SNPs in the region of the LSS gene

The drug target, lanosterol, is synthesised by lanosterol synthase (LSS) (Zhao et al., 2021). A list of SNPs for LSS was obtained through the National Library of Medicine gene database using assembly GRCh37.p13 (PubChem, 2004). The LSS gene is located on chromosome 21 within the base pair region of 47608360 and 47648688. The base pair region was expanded by 5 Kb to identify SNPs between 47603360 and 47653688. The cataract GWAS results were filtered according to the expanded LSS coordinates. Statistical significance was accepted at a multiple testing adjusted *p*-value < (0.05/the number of independent SNPs present in the selected sample). Independent SNPs were identified using PLINK v2.0 to produce a pruned list of variants in approximate linkage equilibrium, using a *r* ^2^ threshold of <0.1, in the LSS gene region.

### Approach 2: comparison of phytosterol-to-lanosterol ratios GWAS with cataract GWAS results

The published GWAS results for the phytosterol-to-lanosterol ratio traits were compared to the generated cataract GWAS results. The phytosterol-to-lanosterol ratio summary statistics were filtered to the GWAS accepted *p*-value < 5 × 10^−8^ and independent SNPs for the look-up analysis were identified for each individual phytosterol-to-lanosterol ratio summary statistic at an *r* ^2^ threshold of <0.1. Independent SNPs were identified using the linkage disequilibrium (LD) pruning function in PLINK v2.0.

The cataract summary statistics were filtered for a *p*-value < (0.05/the number of independent SNPs present across all phytosterol-to-lanosterol traits). Independent SNPs for the multiple testing correction were identified by combining variants across all 8 phytosterol-to-lanosterol traits and LD pruning at an *r* ^2^ threshold of <0.1 using PLINK v2.0.

### Approach 3: genetic risk score analysis

An unweighted genetic risk score (GRS), calculated by the sum of the risk alleles representing decreasing lanosterol, was used to represent a summary of the genetic predisposition for lower levels of lanosterol (Igo et al., 2019; Seral-Cortes et al., 2021). To calculate the GRS for the lanosterol related traits observed in Scholz et al. (2022), we identified independent SNPs by combing all SNPs across the 8 phytosterol-to-lanosterol traits and then pruning the full list variants using an *r* ^2^ threshold of 0.1. Nine independent SNPs were identified across all 8 traits. All effect sizes were set to 1 to perform an unweighted GRS, representing a decreased amount of lanosterol within the ratio. The GRS was generated for all individuals in the UKBB using PLINK v2.0 (Chang et al., 2015). The association between the GRS and cataract outcome was estimated using a logistic regression analysis adjusting for sex, age, agexsex, age squared and the first 10 principal components. Post QC, as described in “Genotyping and imputation of the UKBB”, an additional filter for relatedness was applied, resulting in 36,952 cases and 290,623 controls present in the regression analysis.

## Results

### Case-control description

After QC, 399,020 individuals remained (215,429 Females and 183,591 Males) in the sample. The sample included 45,449 cases and 353,571 controls. Table 1 summarises the sample’s baseline characteristics, including sex, age, BMI and lifetime smoker, diabetes, and employment status at the initial point of assessment.

### Identifying genetic variants in the LSS gene region

**FIGURE 1:**
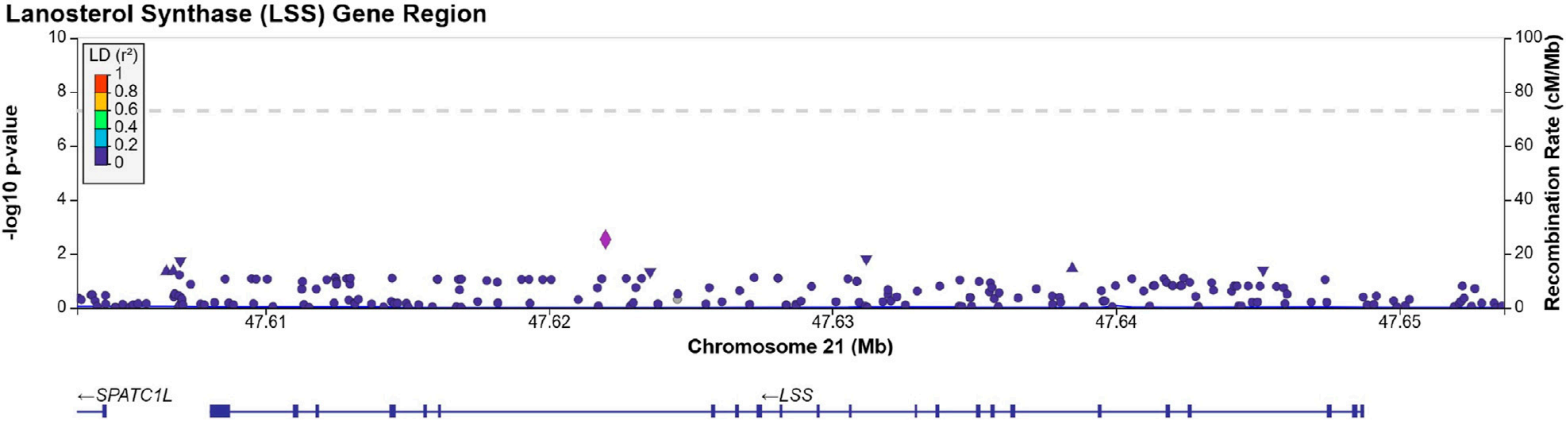
Manhattan plot produced using LocusZoom (Pruim et al., 2010) displaying the results of Supplementary Table S1. The dotted line is set at a significance threshold of 5 × 10^−8^. Left *y*-axis displays -log10 (*p*-value), and *x*-axis displays the LSS base pair region in chromosome 21 (GRCh37).

We identified 203 SNPs available in our summary statistics results and present within the region of the LSS gene. A locus specific Manhattan plot for the 203 SNP can be seen in Figure 1 and full results can be found in Supplementary Table S1. Overall, 13 independent SNPs were identified from the sample of 203 SNPs. SNPs with *p*-value < (0.05/13) were considered statistically significant for affecting cataract outcomes. One SNP, rs191009864, met the significance threshold.

SNP rs191009864 was not present in the replication cohorts. Proxy SNPs, rs137865789, rs147393304, rs141632640 were identified using LD Link (Machiela and Chanock, 2015) in a European population, ±500 Kb of the rs191009864, with an *r* ^2^ = 1.0, and were present in the FinnGen summary statistics, none of the proxy SNPs tested was able to replicate our findings (see Table 2). The proxies identified were not present in the Choquet et al. multi-ethnic GWAS. Approach 1 was repeated in the Choquet et al. multi-ethnic cataract GWAS and no statistically significant SNPs were identified within the LSS gene region. These lookups suggest that the observed association of SNP rs191009864 may represent a false positive.

**TABLE 2:** Description of rs191009864 and proxies (*r* ^2^ = 1.0) located in FinnGen summary statistics.

SNP	Summary statistics	Effect allele	Other allele	EAF	Beta	SE	*p*
rs191009864	REGENIE cataract	A	G	0.013	−0.104	0.035	0.003
rs137865789^a^	FINNGEN cataract senile	A	G	0.004	0.030	0.063	0.630
	FINNGEN cataract other	A	G	0.004	−0.109	0.096	0.258
rs147393304	FINNGEN cataract senile	A	G	0.002	0.044	0.095	0.643
	FINNGEN cataract other	A	G	0.002	−0.138	0.143	0.332
rs141632640^a^	FINNGEN cataract senile	A	G	0.004	0.021	0.061	0.737
	FINNGEN cataract other	A	G	0.004	−0.109	0.093	0.245

^a^ Alleles adjusted to reflect complementary strand.

### Investigating genetic variants across phytosterol-to-lanosterol ratios and cataract GWAS results

The GWAS summary statistics provided by Scholz et al. featured 8 phytosterol-to-lanosterol ratios in the cholesterol synthesis pathway. Using the generally accepted *p*-value threshold of < 5 × 10^−8^ we identified independent SNPs associated with each of the 8 phytosterol-to-lanosterol ratios. We followed-up these SNPs in our cataract GWAS in the UKBB cohort. In total 23 SNPs were identified across both sets of results. The full results of the look-up analysis can be found in Supplementary Table S2.

Within the phytosterol-to-lanosterol ratio summary statistics, we can infer that SNPs with a beta <0 imply an increased presence of lanosterol within the reaction equilibria. For the cataract GWAS, SNPs with a beta <0 indicate a reduced risk of cataracts. The presence of SNPs with a cataract GWAS beta <0 and phytosterol-to-lanosterol beta <0 would indicate increasing lanosterol is protective against cataract risk. Furthermore, SNPs with a cataract GWAS beta >0 and phytosterol-to-lanosterol beta >0 indicates decreased lanosterol is associated to cataract development.

All statistically significant SNPs across all 8 phytosterol-to-lanosterol traits can be tagged by just 9 independent SNPs. Therefore, statistical significance for the cataract associations, adjusted for multiple testing, was set at a *p*-value threshold of < (0.05/9). No statistically significant SNPs were identified to overlap between both cataract and phytosterol-to-lanosterol GWAS results.

### Genetic risk score analysis

We used the identified 9 independent SNPs (rs10205879, rs10208987, rs11057839, rs145288624, rs3846662, rs612169, rs6735229, rs67734975 and rs7599981) to create our unweighted genetic risk score.

The logistic regression for the unweighted genetic risk score on cataract outcomes yielded no statistically significant relationship between individuals’ GRS and cataract risk [OR = 1.002, ln (OR) SE = 0.003, *p* (>|z|) = 0.568].

## Discussion

Current studies surrounding the effect of lanosterol as a treatment for cataract formation are divided. While some studies have supported the effectiveness of lanosterol in treating cataracts (Zhao et al., 2015; Balashova et al., 2018), others have suggested lanosterol is ineffective in the breakdown of cataracts on the lens (Shanmugam et al., 2015; Daszynski et al., 2019). This study aimed to investigate the genetic evidence for an association between lanosterol and cataract to help assess lanosterol as a possible drug treatment option for cataract. Using previously published genetic results for phytosterol-to-lanosterol ratios and generated UKBB genetic data for cataract risk, we tested for genetic evidence around the LSS gene region, compared overlapping genetic variants in GWAS results related to the presence of lanosterol and conducted a genetic risk score analysis. We found the LSS SNP rs191009864 to be statistically significant at a multiple testing adjusted *p*-value < (0.05/13) with risk of cataract in our UK Biobank data. However, we were unable to replicate this, or its identified proxies, in either FinnGen or Choquet et al. summary statistics. Therefore, we cannot claim that robust statistically significant evidence was found to support an association between lanosterol and cataract risk. We did not identify any other SNPs associated with lanosterol metabolism as associated with cataract risk. A score based on these genetic variants also had no evidence of association with cataract risk. Overall, the results of this investigation do not support the use of lanosterol as a treatment for cataract.

Our classification for cataract cases for the cataract GWAS were derived differently than other published results. Various cataract definitions have been used across different studies, for example, Choquet et al. produced a multi-ethnic cataract GWAS using self-reported cataract operation and ICD-10 diagnostic codes (Choquet et al., 2021), whilst an observational study using UKBB cataract cases conducted by Chua et al. used operative codes to define incident cataract (Chua et al., 2021). Our study utilised a combination of cataract definitions, previously used in the UKBB, to maximise available cataract cases. The definition used for the cataract GWAS included operation codes C75.1 Insertion of prosthetic replacement for lens NEC and C71.2 Phacoemulsification of lens from data-field 41272 and the diagnostic codes H25, H26, H28 and Q12.0 from data-field 41270. This definition does not account for the difference in cataract subtypes, for example, cortical, nuclear, or posterior subcapsular. Therefore, our genetic analyses would not account for lanosterol potentially being more effective in treating a specific subtype, a limitation also identified by Chua et al. (2021).

Furthermore, our cataract GWAS was conducted over a European cohort. However, associations between lanosterol and cataract risk have been identified among other ethnic groups. For example, Zou et al. discovered evidence that LSS-rs2968 A allele is associated with nuclear age-related cataract risk within a Chinese population. However, LSS-rs2968 was not found to be statistically significant at a Bonferroni corrected *p*-value within an overall age-related cataract definition (Zou et al., 2020). In our study, LSS-rs2968 also did not reach statistical significance (*p*-value = 0.085) within our cataract GWAS. Additionally, Zou et al. reported a protective effect of LSS-rs2968 A allele, while our results suggest the opposite, indicating a causative effect with cataract. These results suggest the need to investigate the effects of lanosterol on specific cataract subtypes, such as nuclear cataract. Further analysis across additional ethnic groups would also be useful in understanding lanosterol’s potential role as a treatment for cataract.

When generating the UKBB cataract GWAS, REGENIE facilitated a mixed-model-based approach, allowing for the inclusion of related individuals. This provided 45,449 cases alongside 353,371 controls. Another benefit of using REGENIE is that it accounts for the case-control imbalance that was present within the cataract phenotype to reduce the risk of Type 1 errors and inflated estimates, whilst also improving statistical power (Mbatchou et al., 2021).

The effect of lanosterol could also differ depending on the severity and maturity of the cataract. Given that the cataract definition includes operation codes, we can infer the presence of mature cataract cases in the analysis. Considering the severity of cataracts, splitting age-related and early onset cases, and assessing both separately could change the drug efficacy observed. However, surgery can occur due to external factors other than visual impairment and may not be indicative of a cataract endpoint. Furthermore, our genetic analysis assessed the effect of lanosterol on cataract risk through genetic predisposition and should be unaffected by our cataract endpoint.

Using genetics to understand the clinical application of a potential treatment can be difficult but has been successful in recent literature. For example, an investigation on lowering cholesterol levels successfully utilised genetic proxies to mimic the effect of enzyme inhibitors and statins (Ference et al., 2019). A challenge faced in this study was that we considered the exposure to lanosterol over a lifetime at low concentrations, as opposed to the far higher concentrations that would be used in pharmacological interventions. Therefore, we may have observed a lack of association due to a low effect size of lanosterol. However, genetics can still be utilised to ascertain lanosterol’s role in reducing cataract risk using different approaches.

For example, Xu et al., in a review of pharmacotherapy of cataracts, concluded that the use of lanosterol derivatives in steroid eye drops could be more efficient in reversing protein aggregations than lanosterol. As lanosterol is naturally occurring and is a component of the synthesis of cholesterol, it is unable to maintain a high concentration on the lens. Furthermore, lanosterol’s low solubility limits its clinical application. However, Xu et al. found that lanosterol derivatives were able to effectively break down protein aggregations while avoiding the limitations surrounding lanosterol itself (Xu et al., 2020). This suggests that lanosterol derivatives could form a viable cataract treatment, rather than lanosterol itself. Therefore, a genetic analysis of lanosterol derivatives is required to validate its use in cataract prevention.

A further limitation was observing the GWAS results of phytosterol-to-lanosterol ratios rather than using a GWAS on lanosterol levels, due to a lack of data availability. Using the results of the GRS analysis as an example, the independent SNPs used to generate risk scores only indicated the presence of lanosterol within the biosynthesis of cholesterol. A lanosterol levels GWAS is required to analyse the effect of lanosterol itself. Our analysis was conducted on blood measurements of lanosterol and their extension onto the lens. The lens capsule is unique in its selective permeability of macromolecules, and proteins, therefore substances found in the blood may not necessarily reflect what occurs locally on the lens (Danysh and Duncan, 2009). Reyes et al. examined LSS expression levels directly from the lens and discovered its overexpression on cataractous lenses, suggesting potential limitations in assessing blood measurements of lanosterol against cataract risk (Reyes et al., 2023). Expression quantitative trait loci (eQTL) were considered to assess the causal effect of lanosterol on cataract risk. However, current eQTL data is representative of the gene expression of lanosterol in the blood as opposed to the lens. An expansion of eQTL data available would allow for additional genetic analysis.

The results from different genetic analyses found no genetic evidence to support lanosterol’s potential role as a treatment for cataract. Further genetic understanding of the direction of effect of lanosterol levels and its derivatives on cataracts would be beneficial in establishing its role as a non-surgical treatment. Additional analysis across different ethnicities and cataract subtypes may be needed to better understand the effect of lanosterol on cataract risk.

## Data Availability

The data analyzed in this study is subject to the following licenses/restrictions: UK Biobank data are available to all *bona fide* researchers for all types of health-related research which is in the public interest. Requests to access these datasets should be directed to https://www.ukbiobank.ac.uk/enable-your-research/apply-for-access.
