# Supplementary material for: Using genetics to investigate the association between lanosterol and cataract
Source: Front Genet. 2024 Feb 19;15:1231521. doi: 10.3389/fgene.2024.1231521 (PMC10910428; doi:10.3389/fgene.2024.1231521)
Supplement: Supplementary file 1 [file Table1.docx]

**Supplementary Table 1**: SNPs identified in the LSS gene region (expanded by 5Kb) for Approach 1.

| **SNP** | **CHR** | **BP** | **EA** | **OR** | **LOG.OR.SE** | **P** | **EAF** |
| --- | --- | --- | --- | --- | --- | --- | --- |
| rs56293013 | 21 | 47603413 | G | 1.007 | 0.009 | 0.444 | 0.216 |
| rs35960150 | 21 | 47603509 | T | 1.008 | 0.012 | 0.500 | 0.120 |
| rs79044044 | 21 | 47603849 | G | 1.013 | 0.013 | 0.335 | 0.090 |
| rs73144734 | 21 | 47603893 | T | 1.013 | 0.013 | 0.327 | 0.093 |
| rs62215189 | 21 | 47603995 | G | 1.005 | 0.009 | 0.554 | 0.243 |
| rs78331376 | 21 | 47604061 | A | 1.003 | 0.020 | 0.890 | 0.034 |
| rs9647242 | 21 | 47604357 | T | 1.012 | 0.013 | 0.346 | 0.093 |
| rs116170889 | 21 | 47604361 | A | 1.006 | 0.017 | 0.713 | 0.049 |
| rs116442826 | 21 | 47604708 | C | 0.999 | 0.011 | 0.961 | 0.141 |
| rs12482343 | 21 | 47604967 | A | 0.996 | 0.013 | 0.738 | 0.092 |
| rs12482390 | 21 | 47605225 | A | 0.999 | 0.010 | 0.954 | 0.150 |
| rs12482934 | 21 | 47605335 | T | 0.996 | 0.013 | 0.766 | 0.092 |
| rs79937763 | 21 | 47605510 | A | 1.005 | 0.012 | 0.696 | 0.109 |
| rs62212860 | 21 | 47605797 | G | 0.994 | 0.015 | 0.693 | 0.066 |
| 21:47606511_CA_C | 21 | 47606511 | C | 1.015 | 0.007 | 0.044 | 0.531 |
| 21:47606751_TTTTG_T | 21 | 47606751 | T | 1.015 | 0.007 | 0.043 | 0.533 |
| rs118141253 | 21 | 47606769 | C | 1.017 | 0.020 | 0.391 | 0.038 |
| rs8129267 | 21 | 47606797 | A | 0.985 | 0.015 | 0.315 | 0.931 |
| rs8134131 | 21 | 47606805 | T | 0.985 | 0.015 | 0.304 | 0.931 |
| rs148902926 | 21 | 47606935 | G | 0.999 | 0.011 | 0.914 | 0.121 |
| rs183470806 | 21 | 47606955 | T | 0.968 | 0.035 | 0.356 | 0.013 |
| rs142838362 | 21 | 47606966 | T | 0.961 | 0.021 | 0.060 | 0.034 |
| rs146600734 | 21 | 47606986 | C | 1.005 | 0.017 | 0.775 | 0.053 |
| rs182705598 | 21 | 47607000 | C | 0.930 | 0.031 | 0.018 | 0.017 |
| rs570448320 | 21 | 47607056 | T | 0.979 | 0.035 | 0.546 | 0.012 |
| rs9984805 | 21 | 47607057 | G | 1.006 | 0.008 | 0.453 | 0.332 |
| rs28801979 | 21 | 47607088 | A | 1.005 | 0.017 | 0.775 | 0.050 |
| rs118008360 | 21 | 47607364 | T | 1.060 | 0.039 | 0.134 | 0.010 |
| rs555228834 | 21 | 47607713 | ATT | 1.003 | 0.009 | 0.720 | 0.217 |
| rs11702775 | 21 | 47607847 | C | 1.003 | 0.009 | 0.767 | 0.218 |
| rs114033373 | 21 | 47608207 | T | 1.008 | 0.017 | 0.633 | 0.049 |
| rs2968 | 21 | 47608580 | A | 1.013 | 0.007 | 0.085 | 0.572 |
| rs76408279 | 21 | 47608710 | T | 1.008 | 0.017 | 0.661 | 0.049 |
| rs56173768 | 21 | 47608877 | A | 1.003 | 0.009 | 0.772 | 0.216 |
| rs9717 | 21 | 47609510 | C | 1.014 | 0.008 | 0.083 | 0.642 |
| rs118184861 | 21 | 47609578 | G | 1.012 | 0.031 | 0.699 | 0.014 |
| rs914247 | 21 | 47609677 | G | 1.013 | 0.008 | 0.085 | 0.633 |
| rs2330408 | 21 | 47610066 | C | 1.013 | 0.008 | 0.086 | 0.634 |
| rs201035928 | 21 | 47610269 | GGCATGGG  GCTCCCT | 0.998 | 0.015 | 0.896 | 0.061 |
| rs73144744 | 21 | 47611285 | C | 1.014 | 0.011 | 0.202 | 0.144 |
| rs2187118 | 21 | 47611310 | G | 1.012 | 0.008 | 0.106 | 0.573 |
| rs2187119 | 21 | 47611358 | G | 1.003 | 0.009 | 0.748 | 0.217 |
| rs76715041 | 21 | 47611545 | A | 1.001 | 0.023 | 0.966 | 0.026 |
| rs17293705 | 21 | 47611799 | A | 1.035 | 0.027 | 0.201 | 0.019 |
| rs2839139 | 21 | 47612166 | C | 1.013 | 0.008 | 0.090 | 0.634 |
| rs116474329 | 21 | 47612424 | A | 1.008 | 0.017 | 0.661 | 0.049 |
| rs2187120 | 21 | 47612466 | G | 1.013 | 0.008 | 0.077 | 0.573 |
| rs2187121 | 21 | 47612471 | C | 1.013 | 0.008 | 0.077 | 0.573 |
| rs146297977 | 21 | 47612512 | CTG | 1.012 | 0.008 | 0.111 | 0.627 |
| rs73908575 | 21 | 47612512 | G | 1.042 | 0.027 | 0.130 | 0.020 |
| rs3902367 | 21 | 47612867 | G | 1.014 | 0.008 | 0.082 | 0.642 |
| rs372958158 | 21 | 47612941 | C | 0.987 | 0.020 | 0.513 | 0.037 |
| rs561628345 | 21 | 47612986 | A | 0.960 | 0.027 | 0.131 | 0.024 |
| rs2187122 | 21 | 47613004 | C | 1.014 | 0.008 | 0.079 | 0.639 |
| rs10686231 | 21 | 47613160 | GTGTA | 1.006 | 0.013 | 0.648 | 0.104 |
| rs58557992 | 21 | 47613161 | T | 1.004 | 0.011 | 0.710 | 0.129 |
| 21:47613285_ATG_A | 21 | 47613285 | A | 1.011 | 0.015 | 0.484 | 0.071 |
| rs144693442 | 21 | 47613749 | A | 1.006 | 0.017 | 0.716 | 0.049 |
| rs79223895 | 21 | 47614169 | C | 0.999 | 0.015 | 0.930 | 0.061 |
| rs2254522 | 21 | 47614443 | G | 1.005 | 0.010 | 0.572 | 0.180 |
| rs2254524 | 21 | 47614469 | C | 1.014 | 0.008 | 0.079 | 0.642 |
| rs35785446 | 21 | 47614553 | A | 1.007 | 0.017 | 0.686 | 0.049 |
| rs2277824 | 21 | 47614660 | T | 1.005 | 0.011 | 0.666 | 0.130 |
| rs9981910 | 21 | 47614975 | T | 1.005 | 0.011 | 0.679 | 0.130 |
| rs76224955 | 21 | 47615038 | A | 0.998 | 0.022 | 0.912 | 0.028 |
| rs12483507 | 21 | 47615403 | C | 0.992 | 0.026 | 0.764 | 0.022 |
| rs146843341 | 21 | 47615891 | A | 0.998 | 0.022 | 0.921 | 0.032 |
| rs7282841 | 21 | 47616071 | C | 1.013 | 0.008 | 0.087 | 0.633 |
| rs2839140 | 21 | 47616080 | A | 1.013 | 0.007 | 0.086 | 0.572 |
| rs76723404 | 21 | 47616737 | G | 0.998 | 0.015 | 0.913 | 0.061 |
| rs9979525 | 21 | 47616818 | C | 1.013 | 0.008 | 0.087 | 0.634 |
| rs55870069 | 21 | 47616850 | A | 1.016 | 0.013 | 0.216 | 0.095 |
| rs78273090 | 21 | 47616905 | A | 0.999 | 0.015 | 0.955 | 0.061 |
| rs9976233 | 21 | 47616913 | A | 1.013 | 0.007 | 0.085 | 0.572 |
| rs11909555 | 21 | 47617489 | G | 1.005 | 0.010 | 0.582 | 0.180 |
| rs10854480 | 21 | 47617810 | C | 0.987 | 0.008 | 0.098 | 0.363 |
| 21:47618181_TA_T | 21 | 47618181 | T | 1.012 | 0.008 | 0.111 | 0.633 |
| rs115295783 | 21 | 47618203 | C | 1.008 | 0.017 | 0.656 | 0.049 |
| rs7280110 | 21 | 47619039 | A | 1.013 | 0.007 | 0.087 | 0.572 |
| rs4819213 | 21 | 47619300 | A | 1.013 | 0.007 | 0.087 | 0.572 |
| rs117106063 | 21 | 47619333 | T | 1.007 | 0.017 | 0.680 | 0.049 |
| rs4819214 | 21 | 47619784 | A | 1.013 | 0.008 | 0.089 | 0.633 |
| rs2839141 | 21 | 47620082 | A | 1.013 | 0.008 | 0.089 | 0.633 |
| rs28560443 | 21 | 47621036 | C | 1.012 | 0.017 | 0.494 | 0.051 |
| rs797014314 | 21 | 47621715 | C | 0.989 | 0.008 | 0.180 | 0.561 |
| rs117393766 | 21 | 47621751 | A | 0.997 | 0.015 | 0.851 | 0.061 |
| rs62212862 | 21 | 47621869 | A | 0.987 | 0.008 | 0.083 | 0.358 |
| rs191009864 | 21 | 47622000 | A | 0.901 | 0.035 | 0.003 | 0.013 |
| rs55689527 | 21 | 47622727 | G | 0.987 | 0.007 | 0.081 | 0.428 |
| rs2839142 | 21 | 47622870 | C | 1.002 | 0.009 | 0.859 | 0.219 |
| rs7282352 | 21 | 47622981 | C | 0.995 | 0.011 | 0.645 | 0.869 |
| rs11702145 | 21 | 47623068 | G | 1.013 | 0.010 | 0.177 | 0.175 |
| rs4818828 | 21 | 47623274 | G | 0.987 | 0.008 | 0.081 | 0.358 |
| rs4819215 | 21 | 47623573 | A | 0.985 | 0.007 | 0.047 | 0.433 |
| rs34105866 | 21 | 47623848 | G | 0.996 | 0.011 | 0.710 | 0.870 |
| rs771351927 | 21 | 47624536 | CAAAAA | 0.989 | 0.016 | 0.488 | 0.058 |
| rs114334512 | 21 | 47624548 | G | 1.019 | 0.018 | 0.304 | 0.044 |
| rs2075906 | 21 | 47625544 | T | 0.996 | 0.011 | 0.713 | 0.870 |
| rs2839143 | 21 | 47625658 | C | 1.013 | 0.010 | 0.179 | 0.175 |
| rs76660727 | 21 | 47626104 | A | 0.992 | 0.015 | 0.592 | 0.066 |
| rs16978976 | 21 | 47626728 | T | 1.015 | 0.013 | 0.231 | 0.096 |
| rs117330398 | 21 | 47627090 | A | 1.011 | 0.032 | 0.737 | 0.014 |
| rs2839144 | 21 | 47627245 | A | 0.986 | 0.008 | 0.076 | 0.366 |
| rs140139047 | 21 | 47628082 | T | 1.068 | 0.038 | 0.079 | 0.011 |
| rs73144751 | 21 | 47628375 | T | 1.003 | 0.009 | 0.779 | 0.217 |
| rs11089053 | 21 | 47628711 | G | 0.996 | 0.011 | 0.735 | 0.871 |
| rs148436940 | 21 | 47628715 | C | 0.995 | 0.036 | 0.882 | 0.012 |
| rs78155037 | 21 | 47628894 | C | 0.991 | 0.015 | 0.557 | 0.067 |
| rs2839145 | 21 | 47629268 | A | 0.989 | 0.008 | 0.161 | 0.586 |
| rs138130258 | 21 | 47630129 | A | 0.992 | 0.015 | 0.589 | 0.066 |
| rs2839146 | 21 | 47630550 | T | 0.987 | 0.008 | 0.084 | 0.358 |
| rs4819216 | 21 | 47630862 | A | 0.987 | 0.008 | 0.105 | 0.359 |
| rs2839147 | 21 | 47630951 | C | 0.994 | 0.011 | 0.620 | 0.873 |
| rs140233907 | 21 | 47631165 | C | 1.005 | 0.030 | 0.859 | 0.017 |
| rs2001809 | 21 | 47631199 | T | 0.981 | 0.008 | 0.015 | 0.357 |
| rs2009213 | 21 | 47631245 | G | 1.000 | 0.009 | 0.977 | 0.758 |
| rs78276120 | 21 | 47631784 | G | 0.992 | 0.015 | 0.587 | 0.068 |
| rs370233614 | 21 | 47631961 | A | 1.018 | 0.018 | 0.315 | 0.044 |
| 21:47631963_TGG  GCAGGGAG_T | 21 | 47631963 | T | 0.991 | 0.008 | 0.213 | 0.409 |
| rs117773065 | 21 | 47632067 | G | 0.991 | 0.015 | 0.555 | 0.068 |
| rs2839148 | 21 | 47632276 | A | 1.012 | 0.014 | 0.390 | 0.078 |
| rs2839149 | 21 | 47632580 | T | 1.003 | 0.011 | 0.783 | 0.120 |
| rs2839151 | 21 | 47632995 | C | 1.015 | 0.013 | 0.239 | 0.096 |
| rs9974665 | 21 | 47633789 | G | 1.011 | 0.008 | 0.157 | 0.394 |
| rs11701000 | 21 | 47634477 | A | 1.002 | 0.009 | 0.863 | 0.221 |
| rs4239841 | 21 | 47634499 | C | 0.884 | 0.073 | 0.092 | 0.997 |
| rs914248 | 21 | 47634572 | G | 0.999 | 0.011 | 0.900 | 0.872 |
| 21:47634860_GT_G | 21 | 47634860 | G | 1.011 | 0.014 | 0.416 | 0.077 |
| rs536505576 | 21 | 47634862 | A | 1.011 | 0.014 | 0.416 | 0.077 |
| rs73144753 | 21 | 47634915 | A | 1.001 | 0.009 | 0.873 | 0.221 |
| rs34115287 | 21 | 47635176 | C | 1.016 | 0.010 | 0.106 | 0.174 |
| rs60322177 | 21 | 47635545 | A | 1.021 | 0.018 | 0.257 | 0.044 |
| rs2839152 | 21 | 47635577 | T | 0.976 | 0.016 | 0.117 | 0.067 |
| rs9980968 | 21 | 47635627 | G | 1.010 | 0.008 | 0.176 | 0.396 |
| rs11701729 | 21 | 47635713 | A | 0.994 | 0.008 | 0.460 | 0.722 |
| rs2839153 | 21 | 47635856 | G | 0.999 | 0.011 | 0.894 | 0.872 |
| rs73386515 | 21 | 47635883 | T | 1.020 | 0.018 | 0.276 | 0.044 |
| rs76489504 | 21 | 47636557 | A | 1.011 | 0.014 | 0.431 | 0.077 |
| rs141367193 | 21 | 47637197 | ACTGTAGGT | 0.990 | 0.008 | 0.197 | 0.335 |
| rs2839154 | 21 | 47637760 | T | 1.007 | 0.007 | 0.365 | 0.444 |
| rs61591722 | 21 | 47637767 | C | 1.004 | 0.011 | 0.734 | 0.121 |
| rs34625510 | 21 | 47638019 | T | 1.012 | 0.014 | 0.408 | 0.077 |
| rs202056351 | 21 | 47638053 | AC | 0.992 | 0.015 | 0.617 | 0.065 |
| rs9984986 | 21 | 47638463 | G | 1.017 | 0.008 | 0.035 | 0.680 |
| rs11702846 | 21 | 47638872 | T | 1.001 | 0.009 | 0.914 | 0.221 |
| rs2277826 | 21 | 47639492 | G | 1.009 | 0.008 | 0.233 | 0.590 |
| rs74328331 | 21 | 47639548 | T | 0.991 | 0.015 | 0.555 | 0.068 |
| rs117110314 | 21 | 47639614 | C | 1.023 | 0.038 | 0.552 | 0.010 |
| rs73144754 | 21 | 47639876 | A | 1.001 | 0.009 | 0.915 | 0.221 |
| rs2839155 | 21 | 47639992 | A | 1.011 | 0.007 | 0.152 | 0.522 |
| rs6518278 | 21 | 47640571 | G | 1.014 | 0.008 | 0.083 | 0.643 |
| rs35679325 | 21 | 47640980 | G | 1.009 | 0.008 | 0.237 | 0.590 |
| rs2839156 | 21 | 47641196 | A | 1.002 | 0.011 | 0.866 | 0.128 |
| rs13049175 | 21 | 47641326 | A | 1.011 | 0.007 | 0.152 | 0.522 |
| rs13046451 | 21 | 47641373 | A | 1.011 | 0.007 | 0.151 | 0.522 |
| rs2839157 | 21 | 47641700 | T | 1.014 | 0.008 | 0.083 | 0.643 |
| rs2839158 | 21 | 47641794 | T | 1.016 | 0.010 | 0.115 | 0.173 |
| rs2280959 | 21 | 47641996 | G | 0.989 | 0.007 | 0.152 | 0.478 |
| rs2280958 | 21 | 47642016 | T | 0.989 | 0.007 | 0.152 | 0.478 |
| rs2280957 | 21 | 47642272 | T | 0.989 | 0.007 | 0.152 | 0.478 |
| rs2280956 | 21 | 47642323 | G | 0.989 | 0.007 | 0.151 | 0.478 |
| rs2280955 | 21 | 47642397 | T | 0.986 | 0.008 | 0.078 | 0.357 |
| rs11558754 | 21 | 47642609 | A | 1.016 | 0.010 | 0.114 | 0.173 |
| rs117806396 | 21 | 47642817 | T | 1.017 | 0.019 | 0.378 | 0.038 |
| rs11909228 | 21 | 47642914 | T | 0.998 | 0.011 | 0.878 | 0.872 |
| rs73144762 | 21 | 47643382 | G | 1.015 | 0.010 | 0.117 | 0.173 |
| rs12151996 | 21 | 47643442 | A | 1.009 | 0.008 | 0.223 | 0.397 |
| rs4819217 | 21 | 47644092 | T | 0.991 | 0.008 | 0.243 | 0.410 |
| rs4819218 | 21 | 47644169 | T | 0.990 | 0.007 | 0.155 | 0.478 |
| rs6518282 | 21 | 47644279 | A | 0.990 | 0.007 | 0.155 | 0.478 |
| rs6518283 | 21 | 47644334 | T | 0.998 | 0.011 | 0.854 | 0.872 |
| rs6518284 | 21 | 47644428 | G | 0.998 | 0.011 | 0.860 | 0.872 |
| rs6518285 | 21 | 47644667 | C | 0.990 | 0.007 | 0.155 | 0.478 |
| rs200769143 | 21 | 47644808 | GA | 0.993 | 0.015 | 0.619 | 0.068 |
| rs139806871 | 21 | 47644999 | C | 0.992 | 0.015 | 0.605 | 0.068 |
| rs12152059 | 21 | 47645087 | G | 1.039 | 0.027 | 0.157 | 0.020 |
| rs148982990 | 21 | 47645196 | C | 0.941 | 0.029 | 0.040 | 0.018 |
| rs13052806 | 21 | 47645670 | G | 0.990 | 0.007 | 0.162 | 0.478 |
| rs56333186 | 21 | 47645920 | A | 1.017 | 0.013 | 0.181 | 0.096 |
| rs9984242 | 21 | 47645970 | C | 0.997 | 0.007 | 0.693 | 0.506 |
| rs13052767 | 21 | 47646041 | T | 0.967 | 0.033 | 0.309 | 0.014 |
| rs116885460 | 21 | 47646896 | T | 0.993 | 0.015 | 0.613 | 0.068 |
| rs999691 | 21 | 47647382 | A | 0.987 | 0.008 | 0.089 | 0.357 |
| rs76428496 | 21 | 47647439 | A | 0.993 | 0.015 | 0.619 | 0.068 |
| 21:47648729_CCC  CGCCCCT_C | 21 | 47648729 | C | 0.986 | 0.017 | 0.398 | 0.947 |
| rs915803 | 21 | 47648872 | G | 0.998 | 0.007 | 0.771 | 0.498 |
| rs576793892 | 21 | 47649103 | AGGGCGG | 0.996 | 0.011 | 0.739 | 0.165 |
| rs567709402 | 21 | 47649193 | C | 0.967 | 0.037 | 0.362 | 0.012 |
| rs915804 | 21 | 47649802 | C | 0.996 | 0.007 | 0.547 | 0.565 |
| rs73144764 | 21 | 47649917 | T | 0.999 | 0.009 | 0.934 | 0.219 |
| 21:47650214_GT_G | 21 | 47650214 | G | 0.997 | 0.011 | 0.814 | 0.872 |
| rs2298694 | 21 | 47650362 | T | 1.010 | 0.014 | 0.486 | 0.076 |
| rs2839159 | 21 | 47651943 | C | 0.999 | 0.009 | 0.897 | 0.225 |
| rs75300582 | 21 | 47652120 | T | 0.993 | 0.015 | 0.666 | 0.063 |
| rs2839160 | 21 | 47652185 | T | 0.996 | 0.007 | 0.561 | 0.561 |
| rs2839161 | 21 | 47652228 | A | 0.989 | 0.008 | 0.155 | 0.340 |
| rs75159209 | 21 | 47652285 | T | 1.010 | 0.013 | 0.437 | 0.090 |
| rs2839162 | 21 | 47652549 | G | 0.998 | 0.012 | 0.871 | 0.885 |
| rs548916783 | 21 | 47652668 | CA | 1.016 | 0.012 | 0.193 | 0.112 |
| rs79066226 | 21 | 47652906 | T | 0.993 | 0.015 | 0.666 | 0.063 |
| rs17183473 | 21 | 47653345 | G | 0.993 | 0.015 | 0.666 | 0.063 |
| rs8133857 | 21 | 47653422 | C | 1.000 | 0.011 | 0.997 | 0.881 |
| 21:47653623_CAG_C | 21 | 47653623 | C | 0.997 | 0.014 | 0.850 | 0.073 |
