# Supplementary material for: Using genetics to investigate the association between lanosterol and cataract
Source: Front Genet. 2024 Feb 19;15:1231521. doi: 10.3389/fgene.2024.1231521 (PMC10910428; doi:10.3389/fgene.2024.1231521)
Supplement: Supplementary file 2 [file Table2.docx]

**Supplementary Table 2:** Full results of Approach 2. List of SNPs present in cataract GWAS and statistically significant and independent in each phytosterol-to-lanosterol ratio summary statistic at p-value < 5x10^-8^ and r^2^ threshold of < 0.1, respectively. Heatmap of SNP’s phytosterol-to-lanosterol ratio summary statistic betas where green represents beta > 0 and red represents beta < 0. SNPs have been ordered with respect to P-value in Cataract GWAS.

|  |  |  |  |  | **Phytosterol-to-Lanosterol Ratio Pairings and Corresponding Betas** | | | | | | | | |
| --- | --- | --- | --- | --- | --- | --- | --- | --- | --- | --- | --- | --- | --- |
| **SNP** | **CHR** | **Effect Allele** | **Beta in Cataract GWAS** | **P-value in Cataract GWAS** | brf_laf | brt_laf | caf_laf | cat_laf | sif_laf | sit_laf | stf_laf | stt_laf |  |
| rs612169 | 9 | G | 0.021 | 0.008 | NA | NA | NA | 0.047 | NA | NA | NA | NA |  |
| rs550057 | 9 | T | 0.019 | 0.023 | NA | NA | 0.058 | NA | 0.060 | 0.058 | NA | NA |  |
| rs17424122 | 2 | A | -0.034 | 0.028 | NA | NA | NA | 0.120 | NA | NA | NA | NA |  |
| rs111559090 | 2 | A | -0.019 | 0.059 | NA | NA | 0.067 | NA | NA | NA | 0.060 | NA |  |
| rs4076834 | 2 | G | -0.028 | 0.066 | NA | -0.232 | NA | -0.210 | NA | NA | NA | NA |  |
| rs60668987 | 2 | A | -0.039 | 0.070 | NA | NA | NA | NA | NA | NA | NA | -0.273 |  |
| rs10208987 | 2 | G | 0.023 | 0.092 | NA | NA | NA | NA | NA | -0.102 | NA | NA |  |
| rs8302 | 2 | C | -0.014 | 0.144 | 0.074 | 0.069 | NA | 0.064 | 0.079 | NA | NA | NA |  |
| rs6735229 | 2 | C | -0.010 | 0.167 | -0.081 | -0.082 | -0.061 | -0.075 | -0.088 | -0.098 | -0.060 | NA |  |
| rs77370416 | 2 | C | 0.023 | 0.197 | 0.133 | 0.157 | NA | NA | NA | NA | NA | NA |  |
| rs13427362 | 2 | G | 0.017 | 0.246 | NA | NA | NA | NA | -0.103 | NA | NA | NA |  |
| rs3846662 | 5 | G | -0.008 | 0.316 | -0.050 | -0.050 | -0.047 | -0.047 | NA | NA | NA | NA |  |
| rs10205879 | 2 | C | -0.008 | 0.353 | NA | NA | NA | NA | NA | 0.067 | NA | NA |  |
| rs12916 | 5 | C | -0.005 | 0.495 | NA | NA | NA | NA | NA | NA | NA | -0.047 |  |
| rs11057839 | 12 | T | 0.006 | 0.605 | NA | NA | NA | NA | NA | 0.070 | NA | NA |  |
| rs67734975 | 2 | G | -0.009 | 0.608 | NA | NA | NA | NA | 0.125 | 0.135 | NA | NA |  |
| rs7598542 | 2 | C | 0.005 | 0.612 | NA | NA | NA | NA | NA | NA | NA | -0.082 |  |
| rs7599981 | 2 | G | -0.004 | 0.660 | NA | -0.056 | NA | NA | NA | NA | NA | NA |  |
| rs10070119 | 5 | T | -0.003 | 0.664 | NA | NA | NA | NA | NA | -0.053 | NA | NA |  |
| rs145288624 | 2 | T | -0.005 | 0.718 | NA | 0.107 | NA | NA | NA | 0.116 | NA | NA |  |
| rs7590687 | 2 | C | 0.005 | 0.749 | NA | NA | NA | -0.134 | NA | NA | NA | -0.097 |  |
| rs138958276 | 2 | A | 0.004 | 0.814 | NA | NA | NA | NA | NA | NA | NA | -0.123 |  |
| rs140488605 | 2 | T | -0.004 | 0.817 | NA | NA | NA | 0.149 | NA | NA | NA | 0.133 |  |
